# Systemic sclerosis medications and risk of scleroderma renal crisis

**DOI:** 10.1186/s12882-019-1467-y

**Published:** 2019-07-25

**Authors:** S. M. Gordon, J. B. Hughes, R. Nee, R. S. Stitt, W. T. Bailey, D. J. Little, J. D. Edison, S. W. Olson

**Affiliations:** 1 Nephrology Department, Walter Reed National Military Medical Center, 8901 Rockville Pike, Bethesda, MD 20889 USA; 2 Department of Medicine, Walter Reed National Military Medical Center, 8901 Rockville Pike, Bethesda, USA; 3 Rheumatology Department, Walter Reed National Military Medical Center, 8901 Rockville Pike, Bethesda, USA

**Keywords:** Systemic sclerosis, Scleroderma renal crisis, Risk factors, ACE inhibitor, proteinuria

## Abstract

**Background:**

Scleroderma Renal Crisis (SRC) is associated with significant morbidity and mortality. While prednisone is strongly associated with SRC, there are no previous large cohort studies that have evaluated ace inhibitor (ACEi) calcium channel blocker (CCB), angiotensin receptor blocker (ARB), endothelin receptor blocker (ERB), non-steroidal anti-inflammatory drug (NSAID), fluticasone, or mycophenolate mofetil (MMF) use in systemic sclerosis (SSc) and the risk of SRC.

**Methods:**

In this retrospective cohort study of the entire military electronic medical record between 2005 and 2016, we compared the use of ACEi, ARB, CCB, NSAID, ERB, fluticasone, and MMF after SSc diagnosis for 31 cases who subsequently developed SRC to 322 SSc without SRC disease controls.

**Results:**

ACEi was associated with an increased risk for SRC adjusted for age, race, and prednisone use [odds ratio (OR) 4.1, 95% confidence interval (CI) 1.6–10.2, *P* = 0.003]. On stratified analyses, ACEi was only associated with SRC in the presence [OR 5.3, 95% CI 1.1–29.2, *p* = 0.03], and not the absence of proteinuria. In addition, a doubling of ACEi dose [61% vs. 12%, *p* < 0.001) and achieving maximum ACEi dose [45% vs. 4%, p < 0.001] after SSc diagnosis was associated with future SRC. CCB, ARB, NSAIDs, ERB, fluticasone, and MMF use were not significantly associated with SRC.

**Conclusion:**

ACEi use at SSC diagnosis was associated with an increased risk for SRC. Results suggest that it may be a passive marker of known SRC risk factors, such as proteinuria, or evolving disease. SSC patients that require ACEi should be more closely monitored for SRC.

## Background

Scleroderma Renal Crisis (SRC) develops abruptly in systemic sclerosis (SSc) and is associated with significant morbidity and mortality [1–4]. Proteinuria, anemia, thrombocytopenia, elevated erythrocyte sedimentation rate (ESR), chronic hypertension, chronic kidney disease, prednisone use, and diffuse cutaneous involvement at SSc diagnosis are associated with future SRC [1–5]. High risk SSc subjects could benefit from treatments that prevent SRC; however, medications already used in SSc warrant initial investigation. Due to the successful treatment of SRC, Angiotensin Converting Enzyme inhibitors (ACEi) were initially thought to be a good candidate for prophylactic therapy [6–8]. But, previous case series and small cohort studies found no benefit for initiation of an ACEi at SSc diagnosis to prevent SRC [9–12]. Some studies even reported a potential paradoxical association with incidence and severity of SRC [13–16]. Angiotensin Receptor Blockers (ARB) have not been robustly evaluated independent from ACEi. Calcium channel blockers (CCB) are a consensus second line agent in SRC [17]. One small cohort study found that calcium channel blockers may prevent SRC, but this finding has not been confirmed by a larger cohort study [18]. Circulating endothelin-1 (ET-1) is elevated and arteriolar ET-1 staining is prominent at SRC diagnosis [19]. In addition Endothelin receptor blockers (ERB) may improve SRC outcomes [20–22]. Therefore ERBs are attractive candidates to prevent SRC after SSc diagnosis, but have not been previously studied. Mycophenolate mofetil (MMF) is efficacious for diffuse cutaneous disease and interstitial lung disease in SSc but has not been evaluated at or before SRC [23–25]. Conversely, it is important to identify any common medications used by SSc patients that may increase risk of SRC. Systemic steroids are associated with SRC but nasal steroids have not been studied [9]. Non-steroidal anti-inflammatory drugs (NSAIDs) use is associated with both hypertension and acute kidney injury (AKI) due to afferent arterial constriction, but any correlation to SRC in SSc is unknown. We aim to evaluate the effect of commonly used medications in SSC on the subsequent risk of SRC.

## Methods

**Fig. 1:**
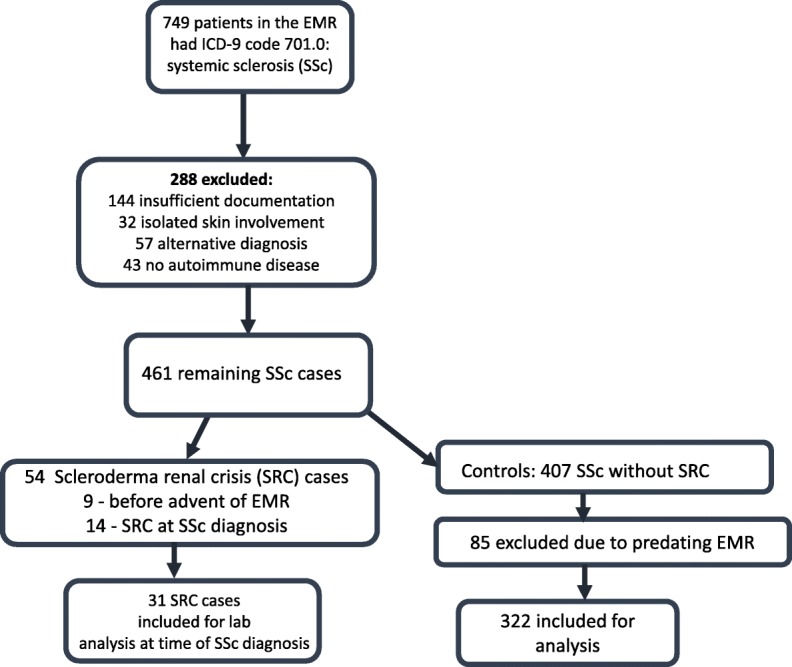
A flow diagram for the identification of Systemic Sclerosis and Scleroderma Renal Crisis cases

We conducted a retrospective systemic sclerosis cohort study of medications and the risk of future SRC. The Military Health System (MHS) electronic medical record (EMR) was queried for systemic sclerosis between the years 2005–2016 using the ICD-9 code 710.1 which identified 749 potential cases. A comprehensive EMR review was performed for each potential case to confirm diagnosis and collect background data with a detailed process previously reported (5, Fig. 1). There were 353 cases with confirmed SSc. SSc was defined by meeting classification criteria of either the American College of Rheumatology (ACR) or the European League Against Rheumatism (EULAR) 2013 classification as determined by a rheumatologist. The MHS is a global medical network within the U.S Department of Defense consisting of approximately 9.6 million active/retired service members and their families. It is a closed health system consisting of approximately 65 hospitals and 412 clinics spanning the world which share a common EMR [26, 27]. The MHS provides longitudinal follow up of a diverse population. There were 31 cases of SRC identified in the SSc cohort. SRC was defined by at least one of the following criteria in the absence of another clinical explanation for AKI and/or hypertensive emergency: 1. AKI requiring renal replacement therapy (RRT) 2. A doubling of serum creatinine. 3. A 50% rise in serum creatinine with new onset hypertension (blood pressure greater than or equal to 140/90 mmHg). 4. Hypertensive urgency or emergency defined by an abrupt onset of BP ≥180/110 mmHg requiring hospitalization or evidence of end organ damage. Essential hypertension and chronic kidney disease (CKD) present at SSc diagnosis was confirmed by comparison data up to 2 years before SSc diagnosis. In addition to the previously published data [5], for each SRC case, we recorded ACE inhibitor, ARB, CCB, ERB, MMF, fluticasone, and NSAID use and their doses between SSc and SRC diagnosis. For comparison, the same information was collected for each SSc without SRC disease control between SSc diagnosis and 5 years after diagnosis. For anti-hypertensive medications, in addition to medication use, doubling of medication dose and achievement of maximum dose during the defined follow up period were also primary outcomes. For the purposes of this study, maximum dose was defined as equal to, or greater than, the following daily doses: Ramipril 10 mg, Lisinopril 40 mg, benazopril 40 mg, fosinopril 40 mg, Telmisartan 80 mg, Irbesartan 300 mg, Losartan 100 mg, Valsartan 320 mg, Candesartan 32 mg, Adalat 90 mg, amlodipine 10 mg, felodipine 10 mg, Diltiazem 360 mg and Verapamil 360 mg daily. Doubling of anti-hypertension medication dose was based on change over time in relationship to the initial dose at or following SSc diagnosis.

Analyses were performed using Stata 14 SE (Stata Corp, College Station, TX). Incidence of primary outcomes were compared between SRC cases and SSc without SRC disease controls using univariate analyses performed with Chi-square testing for categorical variables (Fisher’s exact test used for violations of Cochran’s assumptions). We then conducted logistic regression analyses in forward stepwise fashion to evaluate the association between ACEi and the risk of SRC. Due to sample size limitations, not all significant variables in univariate analysis could be included in an individual regression model. Significant variables in the univariate analysis were therefore evaluated in separate models. All models included significant demographic covariables in addition to unique variables. One model included prednisone as the only other medication previously shown to be associated with SRC. Because thrombotic microangiopathy (TMA) is associated with SRC, a second model included anemia and thrombocytopenia. A third model included variables associated with significant systemic disease to include cardiac involvement, pulmonary hypertension, and an elevated ESR. A fourth model included CKD as a covariate. A final model included the anti-RNAPOL3 and anti-RO antibodies which are the only antibodies known to be associated with SRC. We then stratified the logistic regression models based on presence/absence of proteinuria. Specifically, in the subgroup of patients with proteinuria, the model only had 34 observations. We therefore used exact logistic regression which provides more reliable parameter estimates in small samples than does standard logistic regression.

The area under the receiver operating characteristic (ROC) curve ranged from 0.81 to 0.88 (*c* statistic) indicating acceptable discrimination of the outcome variable in the individual models. To test model calibration, we calculated the Hosmer-Lemeshow goodness-of-fit test; *p*-values for the models were non-significant indicating no evidence of poor fit.

This study (#418335) was approved by the Walter Reed National Military Medical Center Institutional Review Board (IRB).

## Results

SRC cases were more likely to be older and Black. In addition, they more frequently had cardiac manifestations of SSc and pulmonary hypertension. Proteinuria, anemia, thrombocytopenia, essential hypertension, baseline CKD, elevated ESR, anti-RNAPOL3 antibody, and anti-Ro antibody were also more common in the SRC group than the SSc without SRC group at time of SSc diagnosis (5, Table 1).

**Table 1:** Demographic, clinical, and serologic characteristics of Scleroderma Renal Crisis (SRC) cases and Systemic Sclerosis (SSc) without SRC disease controls

	SRC (*n* = 31)	SSc without SRC (*n* = 322)	*P*-value
Race:			
White	48 (15/31)	57 (158/279)	NS
Black	42 (13/31)	24 (66/279)	0.03
Other	1 (3/31)	20 (63/279)	NS
Sex (%female)	74 (23/31)	84 (271/322)	NS
Age (yrs)	53 (40,60)	46 (37, 54)	0.01
Time Between SSc and SRC (yrs)	3 (1,5)	NA	NA
SSc Follow Up (yrs)	6 (3,8)	5 (2,8)	NS
Pulmonary Fibrosis (%)	42 (13/31)	31 (100/322)	NS
Pulmonary HTN (%)	39 (12/31)	12 (40/322)	< 0.001
Cardiac Involvement (%)	23 (7/31)	5 (17/322)	0.002
GI Involvement (%)	77 (24/31)	82 (265/322)	NS
RNP (%)	90 (28/31)	97 (313/322)	NS
Digital Ulcers (%)	29 (9/31)	23 (73/322)	NS
Prior Prednisone (%)	65 (20/31)	19 (62/322)	< 0.001
Other IST (%)	32 (10/31)	32 (104/322)	NS
Diffuse	39 (12/31)	16 (52/322)	0.004
Limited	61 (19/31)	74 (237/322)	NS
Sine	0 (0/31)	1 (4/322)	NS
Unknown	0 (0/31)	9 (29/322)	NS
ANA:	83 (25/30)	95 (228/242)	0.04
Speckled	41 (9/22)	33 (73/221)	NS
Nucleolar	27 (6/22)	41 (90/221)	NS
Homogeneous	18 (4/22)	16 (36/221)	NS
Centromere	4 (1/22)	9 (20/221)	NS
Other	9 (2/22)	1 (2/221)	NS
RNAPOLIII	50 (7/14)	19 (15/76)	0.02
Centromere	5 (1/17)	52 (100/192)	< 0.001
SCL-70	5 (1/17)	18 (40/183)	NS
U3-RNP	21 (3/14)	28 (11/40)	NS
SSA	35 (9/26)	13 (29/228)	0.006
SSB	8 (2/26)	4 (9/219)	NS

*HTN* Hypertension, *GI* Gastrointestinal, *RNP* Raynaud’s phenomenon, *IST* Immunosuppression therapy

The SRC cases were more frequently prescribed an ACEi between SSc diagnosis and SRC than the SSc without SRC cases during the 5 years after SSc diagnosis [77% vs. 33%, Table 2]. In adjusted analyses, ACEi was a significant predictor for SRC in models that accounted for prednisone use [OR-4.1]; thrombocytopenia and anemia [OR-6.6]; cardiac involvement, pulmonary hypertension and elevated ESR [OR-4.9]; CKD [OR-7.3], and RNAPOL3-Ab and Ro-Ab [OR-7.1] (Table 3). More SRC cases also had a doubling of the ACEi dose [61% vs. 12%] and achieved maximum ACEi dose [45% vs. 4%] between SSc and SRC diagnoses than SSc without SRC cases in the 5 years following SSc diagnosis (Table 2).

**Table 2:** Univerate analysis of medication use, dose, and maximum dose achieved and risk of SRC

	SRC (n = 31)	SSc without SRC (n = 322)	*P*-Value
Medication Use			
ACE Inhibitor	77 (24/31)	33 (108/332)	< 0.001
ARB	13 (4/31)	16 (53/322)	NS
ACE inhibitor or ARB	84 (26/31)	40 (128/322)	< 0.001
CCB (dihydropyridine)	65 (20/31)	66 (214/322)	NS
CCB (non-dihydropyridine)	6 (2/31)	4 (322)	NS
CCB (any)	65 (20/31)	70 (226/322)	NS
Endothelin Receptor Blocker (ERB)	6 (2/31)	3 (9/322)	NS
Mycophenolic Mofetil (MMF)	16 (5/31)	11 (36/322)	NS
Nonsteroidal Anti-inflammatory Drugs (NSAIDs)	84 (26/31)	77 (249/322)	NS
Fluticasone	42 (13/31)	56 (180/322)	NS
Doubling of Dose			
ACE Inhibitor	61 (19/31)	12 (38/322)	*P* < 0.001
ARB	3 (1/31)	3 (10/322)	NS
ACE inhibitor or ARB	61 (19/31)	14 (45/322)	NS
CCB (dihydropyridine)	32 (10/31)	23 (73/322)	NS
CCB (non-dihydropyridine)	0 (0/31)	3 (10/322)	NS
CCB (any)	32 (10/31)	25 (80/322)	NS
Achieve Max Dose			
ACE Inhibitor	45 (14/31)	4 (14/322)	< 0.001
ARB	10 (3/31)	2 (8/322)	0.06
ACE inhibitor or ARB	45 (19/31)	7 (21/322)	< 0.001
CCB (dihydropyridine)	19 (6/31)	16 (50/322)	NS
CCB (non-dihydropyridine)	3 (1/31)	3 (10/322)	NS
CCB (any)	19 (6/31)	16 (52/322)	NS

**Table 3:** ACEi as a predictor for SRC in logistic regression models. All models were adjusted for age and race

Adjusted Models	OR	95% confidence interval	*P*-value
Prednisone	4.1	1.6–10.2	0.003
Anemia, Thrombocytopenia	6.6	2.3–19.7	0.001
Cardiac Involvement, Pulmonary Hypertension, Elevated ESR	4.9	1.7–14.1	0.004
CKD	7.3	2.5–21.2	< 0.001
RNAPOL3-Ab, Ro-Ab	7.1	1.5–33.3	0.01

On stratified analyses there was no signficant association between ACEi and SRC in the absence of proteinuria. But in the presence of proteinuria, ACEi was significantly associated with SRC [OR-5.3] (Table 4).

**Table 4:** ACEi as a predictor for SRC stratified by proteinuria

Adjusted Models^a^	OR	95% confidence interval	*P*-value
Proteinuria	5.3	1.1–29.2	0.03
No Proteinuria	2.1	0.44–10.5	0.348

^a^Adjusted for age, race, and sex

There was no significantly different exposure to ARB, CCB, ERB, MMF, NSAID, or fluticasone between the two groups (Table 2).

## Discussion

In this comprehensive retrospective SSc cohort study we found that ACE inhibitors were associated with future SRC even when adjusted for potential confounding variables, but that ARB, CCB, ERB, NSAIDs, MMF, and fluticasone were not positively or negatively associated with SRC. The association of ACEi treatment in SSc with the development SRC is counterintuitive because it is a proven efficacious treatment for SRC, but previous studies do not contradict this conclusion [6–8]. No previous retrospective SSc case series or cohort demonstrated that ACEi reduced risk of SRC. In fact, up to 25% of SRC cases were on an ACEi prior to diagnosis [9–16]. Some SSc studies reported a trend toward ACEi being associated with SRC, long term dialysis dependence after SRC, and death [13–16]. While there is no definitive explanation for the association between ACEi and SRC in our study, it is unlikely that ACEi is both a cause and treatment for SRC. Our data suggests that ACEi use is likely a surrogate marker for an evolving vasculopathy which culminates in fulminant SRC. ACEi would most likely be used in SSc for hypertension and proteinuria which are both associated with future SRC [5]. Our study showed that ACEi use was associated with SRC in SSc patients with proteinuria, but not those without proteinuria. If ACEi had a direct pathogenic effect, it would be expected to be associated with future SRC in a SSc cohort without proteinuria as well. In addition, we found that the doubling of ACEi dose and achievement of maximum dose were more common in the SSc group that developed SRC. This would be consistent with a dose escalation for progressive hypertension or proteinuria secondary to an evolving or smoldering vasculopathy preceding acute onset of SRC. Proteinuria is a well-established marker of vasculopathy [28–32]. In addition there are previous studies of renal functional reserve, intermediate weight proteinuria, and renal vascular resistive indices in SSc that strongly suggest an evolving subclinical vasculopathy in a subset of patients [28, 33–35]. Of note, the rate of ACEi use in our SSc cohort is higher than previously reported for both SRC and SSc without SRC cases [9–16]. There are multiple potential explanations. The insular nature of the military electronic medical record may have allowed better capture of SSc cases taking ACEi. Because this cohort was more recent that past cohorts, ACEi use may have increased because of more widespread acceptance as a first line anti-hypertensive agent, more stringent blood pressure goals, and more focus on managing proteinuria to improve prognosis. In addition, our cohort has a larger number of men and African Americans who are more prone to essential hypertension and therefore more commonly taking ACEi. Finally, given the clear benefit of ACEi for treatment of SRC, more providers over time may have preferentially selected ACEi for management of hypertension in SSc for potential prophylactic benefits.

We found no evidence that CCB reduced risk of SRC. One previous retrospective cohort study reported that CCB in SSc was associated with a decreased incidence of SRC. But this study compared trimesters of CCB exposure between SRC and SSc without SRC groups instead of the more clinically relevant comparison of the percent of cases in each group who received CCB after SSc diagnosis [18]. In addition, our study did not show a difference in dose escalation or achievement of maximum dose CCB between groups.

We also found no evidence that ERB use in SSc reduced the risk of SRC. While there are multiple case reports and one open labeled pilot study which suggest a treatment benefit at the time of SRC diagnosis, no previous SSc cohort studies evaluated the impact of ERB use on future SRC [20–22]. Our results must be interpreted with caution. The overall use of ERBs in this cohort was low, limiting the ability to determine a statistically significant benefit even if present. Given the mechanism of disease and medication, a more robust study of the potential prophylactic benefit of ERB in at risk SSc patients is warranted.

In addition, we found no evidence that MMF use in SSc was negatively associated with SRC. While MMF has demonstrated therapeutic benefit for diffuse cutaneous disease and pulmonary fibrosis in SSc, efficacy for treatment or prophylaxis of SRC has not been evaluated [23–25].

We hypothesized that fluticasone use in SSc may increase the incidence of SRC. Systemic corticosteroid are strongly associated with SRC risk [9]. The systemic bioavailability of fluticasone is much lower than oral steroids but still present [36]. But in our SSc cohort, fluticasone was not associated with an increased risk of SRC.

Similarly we hypothesized that NSAID use in SSc may increase the incidence of SRC. NSAIDs cause afferent arterial constriction which could further stimulate an already overactive renin-angiotensin-system to trigger SRC. NSAIDs have not been previously evaluated as a risk factor for SRC, potentially because most NSAIDs are over-the-counter medications and difficult to accurately evaluate in retrospective chart reviews. NSAIDs are accurately recorded in the military electronic medical record, because patients can receive the medications at no cost and often pick them up with other prescribed medications. While NSAID use was common in SSc there was no association with future development of SRC.

This study has limitations, many of which are inherent to a retrospective cohort study and were discussed in detail previously [5]. Notably, diagnoses were often based on subspecialty notes that often did not reliably include specific details about diagnostic studies such as diffuse capacity of the lung for carbon monoxide (DLCO), forced vital capacity (FVC), estimated pulmonary artery pressures, capillaroscopy, or modified Rodnan skin score (mRSS). More detailed information in these areas would have allowed for more nuanced data analysis. In addition, our analysis used drug classes. But, there were multiple different medications in each drug class. Though less likely, it is possible that specific medications within each drug class had unique associations with SRC. Analysis of the percent of cases with a doubling of initial dose and achievement of maximum dose was an effort to normalize for different medication types within drug class. Specifically, NSAID use may have been underestimated. Though most people get their NSIADs from military pharmacies, some could have taken over-the-counter NSAIDs that went undocumented. Similar to our previous study, proteinuria was based on urinalysis because patients rarely had proteinuria quantification recorded before SRC diagnosis. Due to limited number of study cases, we were unable to include all potential confounding variables in any individual regression model.

## Conclusions

This study has potential clinical implications. Our data suggests that ACEi use in SSc is most likely a surrogate for other established SRC risk factors such as proteinuria and chronic hypertension, or evolving subclinical SRC and not a cause of SRC. SSc patients requiring dose escalations or on maximum dose ACEi should be monitored very closely for fulminant SCR. Conversely, nasal fluticasone and oral NSAIDs use in SSc appear to confer no additional risk for SRC. There was no clear prophylactic benefit of ERB in this study, but the low overall ERB use precludes definitive conclusions even on associations.

## Data Availability

The datasets used and/or analysed during the current study are available from the corresponding author on reasonable request.

## Abbreviations

ACEi: Ace inhibitor
ACR: American College of Rheumatology
AKI: Acute kidney injury
ARB: Angiotensin receptor blocker
CCB: Calcium channel blocker
CKD: Chronic kidney disease
EMR: Electronic medical record
ERB: Endothelin receptor blocker
ET-1: Endothelin-1
EULAR: European League Against Rheumatism
MHS: The Military Health System
MMF: Mycophenolate mofetil
NSAID: Non-steroidal anti-inflammatory drug
ROC: Receiver operating characteristic
RRT: Renal replacement therapy
SRC: Scleroderma Renal Crisis
SSc: Systemic sclerosis
TMA: Thrombotic microangiopathy

## References

[1] Gabrielli A, Avvedimento EV, Krieg T (2009). Scleroderma. N Engl J Med.

[2] Mouthon L, Bussone G, Berezne A, Noel LH, Guillevin L (2014). Scleroderma Renal Crisis. J Rheumatol.

[3] Shanmugam VK, Steen VD (2012). Renal disease in scleroderma: an update on evaluation, risk stratification, pathogenesis and management. Curr Opin Rheumatol.

[4] Steen VD (2014). Kidney involvement in systemic sclerosis. Presse Med.

[5] Gordon Sarah M., Stitt Rodger S., Nee Robert, Bailey Wayne T., Little Dustin J., Knight Kendral R., Hughes James B., Edison Jess D., Olson Stephen W. (2018). Risk Factors for Future Scleroderma Renal Crisis at Systemic Sclerosis Diagnosis. The Journal of Rheumatology.

[6] Steen VD, Costantino JP, Shapiro AP, Medsger TA (1990). Outcome of renal crisis in systemic sclerosis: relation to availability of angiotensin converting enzyme(ACE) inhibitors. Ann Intern Med.

[7] Beckett VL, Donadio JV, Brennan LA, Conn DL, Osmundson PJ, Chao EY (1985). Use of captopril as early therapy for renal scleroderma: a prospective study. Clin Proc.

[8] Steen VD, Medsger TA (2000). Long-term outcomes of scleroderma renal crisis. Ann Intern Med.

[9] Steen VD, Medsger TA (1998). Case-control study of corticosteroids and other drugs that either precipitate or protect from the development of scleroderma renal crisis. Arthritis Rheum.

[10] Demarco PJ, Weisman MH, Seibold JR, Furst DE, Wong WK, Hurwitz EL (2002). Predictors and outcomes of scleroderma renal crisis. Arthritis Rheum.

[11] Walker JG, Ahern MJ, Smith MD, Coleman M, Pile K, Rischmueller M (2003). Scleroderma renal crisis: poor outcome despite aggressive antihypertensive treatment. Intern Med J.

[12] Codullo V, Cavazzana I, Bonino C, Alpini C, Cavagna L, Cozzi F, Papa ND, Franceschini F, Guiducci S, Morozzi G, Ruffatti A, Ferri C, Giacomelli R, Matucci-Cerninic M, Valentini G, Montecucco C (2009). Serologic profile and mortality rates of scleroderma renal crisis in Italy. J Rheumatol.

[13] Penn H, Howie AJ, Kingdon EJ, Bunn CC, Stratton RJ, Black CM (2007). Scleroderma renal crisis:patient characteristics and long-term outcomes. Q J Med.

[14] Teixeira L, Mouthon L, Mahr A, Berezne A, Agard C, Mehrenberger M (2008). Mortality and risk factors of scleroderma renal crisis: a French retrospective study of 50 patients. Ann Rheum Dis.

[15] Guillevin L, Berezne A, Seror R, Teixeira L, Pourrat J, Mahr A, Hachulla E, Agard C, Cabane J, Vanhille P, Harle JR, Deleveauz I, Mouthon L (2012). Scleroderma renal crisis: a retrospective multicenter study on 91 patients and 427 controls. Rheumatology.

[16] Hudson M, Baron M, Tatibouet S, Furst DE, Khanna D (2014). Exposure to ACE inhibitors prior to the onset of scleroderma renal crisis-results from the International Scleroderma Renal Crisis Survey. Semin Arthritis Rheum.

[17] Walker KM, Pope J (2012). Treatment of systemic sclerosis complications: what to use when first-line treatment fails- a consensus of systemic sclerosis experts. Semin Arthritis Rheum.

[18] Montanelli G, Beretta L, Santaniello A, Scorza R (2013). Effect of dihydropyridine calcium channel blockers and glucocorticoids on the prevention and development of scleroderma renal crisis in and Italian case series. Clin Exp Rheumatol.

[19] Mouthon L, Mehrenberger M, Teixeira L, Fakhouri F, Berezne A, Guillevin L (2011). Endothelin-1 expression in scleroderma renal crisis. Hum Pathol.

[20] Penn H, Quillinan N, Khan K, Chakravarty K, Ong VH, Burns A (2013). Targeting the endothelin axis in scleroderma renal crisis: rationale and feasibility. QJ Med.

[21] Dhaun N, MacIntyre IM, Bellamy COC, Kluth DC (2009). Endothelin receptor antagonism and renin inhibition as treatment options for scleroderma kidney. Am J Kidney Dis.

[22] Izzedine H, Rouvier P, Deray G (2013). Endothelin receptor antagonism-based treatment for scleroderma renal crisis. Am J Kidney Dis.

[23] Mendoza FA, Nagle SJ, Lee JB, Jimenez SA (2012). A prospective observational study of mycophenolate mofetil treatment in progressive diffuse cutaneous systemic sclerosis of recent onset. J Rheumatol.

[24] Tashkin DP, Elashoff R, Clements PJ, Goldin J, Roth MD, Furst DE (2006). Cyclophosphamide versus placebo in scleroderma lung disease. N Engl J Med.

[25] Tashkin DP, Roth MD, Clements PJ, Furst DE, Khanna D, Kleerup EC (2016). Mycophenolate mofetil versus oral cyclophosphamide in scleroderma-related interstitial lung disease (SLS II): a randomized controlled, double-blind, parallel group trial. Lancet Respir Med.

[26] Military Health System and the Defense Health Agency. Military Health System Review Final Report 2014. https://health.mil/About-MHS. Accessed 22 July 2019.

[27] Dorrance KA, Ramchandani S, Neil N, Fisher H (2013). Leveraging the military health system as a laboratory for health care reform. Mil Med.

[28] Shanmugam VK, Steen VD (2010). Renal manifestations in scleroderma: evidence for subclinical renal disease as a marker of vasculopathy. Int J Rheumatol.

[29] Schmieder RE, Mann JFE, Schumacher H, Gao P, Mancia G, Weber MA, McQueen M, Koon T, Yusuf S (2011). Changes in albuminuria predict mortality and morbidity in patients with vascular disease. J Am Soc Nephrol.

[30] de Zeew D, Parving HH, Henning RH (2006). Microalbuminuria as an early marker for cardiovascular disease. J Am Soc Nephrol.

[31] Klausen K, Borch-Johnsen K, Feldt-Rasmussen B, Jensen G, Clausen P, Scharling H (2004). Very low levels of microalbuminuria are associated with increased risk of coronary heart disease and death independently of renal function, hypertension, and diabetes. Circulation.

[32] Hillege HL, Fidler V, Diercks GFH, van Gilst WH, de Zeeuw D, van Veldhuisen DJ (2002). Urinary albumin excretion predicts cardiovascular and noncardiovascular mortality in general population. Circulation.

[33] Livi R, Teghini L, Pignone A, Generini S, Matucci-Cerinic M, Cagnoni M (2002). Renal functional reserve is impaired in patients with systemic sclerosis without clinical signs of kidney involvement. Ann Rheum Dis.

[34] Seilberlich B, Hunzelmann N, Krieg T, Weber M, Shulze-Lohoff E (2008). Intermediate molecular weight proteinuria and albuminuria identify scleroderma patients with increased morbidity. Clin Nephrol.

[35] Rivolta R, Mascagni B, Berruti V, di Palo FQ, Elli A, Scorza R (1996). Renal vascular damage in systemic sclerosis patients without clinical evidence of nephropathy. Arthritis Rheum.

[36] Daley-Yates PT, Baker RC (2001). Systemic bioavailability of fluticasone propionate administered as nasal drops and aqueous nasal spray formulations. Br J Clin Pharmacol.

